# Infant gut microbiota restoration: state of the art

**DOI:** 10.1080/19490976.2022.2118811

**Published:** 2022-09-10

**Authors:** Katri Korpela, Willem M. de Vos

**Affiliations:** a Human Microbiome Research Program, Faculty of Medicine, University of Helsinki, Helsinki, Finland; b Laboratory of Microbiology, Wageningen University, WE Wageningen, The Netherlands

**Keywords:** Intrapartum antibiotics, birth mode, cesarean section, lactic acid bacteria and bifidobacteria, breastfeeding, fecal microbiota transplant, vaginal seeding

## Abstract

The gut microbiota has a central role in the programming of the host’s metabolism and immune function, with both immediate and long-term health consequences. Recent years have witnessed an accumulation of understanding of the process of the colonization and development of the gut microbiota in infants. The natural gut microbiota colonization during birth is frequently disrupted due to C-section birth or intrapartum or postpartum antibiotic exposure, and consequently aberrant gut microbiota development is common. On a positive note, research has shown that restoration of normal gut microbiota development is feasible. We discuss here the current understanding of the infant microbiota, provide an overview of the sources of disturbances, and critically evaluate the evidence on early life gut microbiota restoration for improved health outcomes by analyzing published data from infant gut microbiota restoration studies.

## Microbial colonization of infant gut

One of the most exciting events that takes place in life is the birth of a child. As the human microbiota is known to play an important and determining role in health, it is of great interest to understand how, when and where the newborn is colonized. This notably holds for the intestinal microbiota that rapidly becomes the most dominant microbial part of our body. Early approaches two decades ago using 16S rRNA gene sequence-based molecular tools applied to the fecal microbiota already indicated the existence of succession events in early life, the impact of antibiotics on this process, and the differences between breastfed and formula-fed babies.^1,2^ Present-day high-throughput 16S rRNA and metagenomic approaches have allowed for refining this picture with attention to all microbes, including viruses, providing global views on early life development, and detailing the origin of microbial strains. A wealth of association studies have been reported of microbiota and health and disease, and in some cases studies using fecal microbiota transfer have provided quantum leap insight regarding causality.^3–5^

Microbial colonization, i.e. the permanent establishment of viable and hence multiplying microbial populations on and in the body of the host, is an important process that affects long-term health.^6^ The gut microbiota has a central role in the early-life programming of the host’s metabolism and immune function, with both immediate and long-term health consequences. In the first few years after birth, the overall gut microbiota development follows a biologically determined pattern, which is consistent across cultures and geographic areas.^7^ Due to the specific temporal features of this development, the host receives specific microbial signals at specific age windows. If these signals are altered or missing due to abnormal microbiota development, the host’s physiological development may be affected. Deviations from normal microbiota succession in infants and young children often occur without clear symptoms, but can increase the susceptibility to later health problems, such as allergic disease including asthma,^8–10^ autoimmune diseases such as type 1 diabetes,^11^ and overweight.^12^

Although microbial DNA has been identified in placenta, amniotic fluid and meconium samples,^13–17^ the current evidence-based consensus is that healthy infants are normally colonized by microbes during and after birth, not *in utero*.^18,19^ The infant is exposed to maternal vaginal, fecal and skin microbes during and after birth, depending on the mode of birth. However, bacteria establish only in habitats that provide suitable conditions for them: while vaginal and skin microbes may be transiently detected in infant fecal samples, the infant gut is permanently colonized specifically by gut bacteria that are partly of maternal origin.^20–25^ The neonate gut provides a different environment compared to the maternal gut, and therefore only infant-adapted strains from the mother have the ability to establish permanent populations in the infant. The dominant mother-to-infant transmitted gut microbes include *Bifidobacterium* and *Bacteroides* strains,^24,25^ which have the capacity to utilize human milk oligosaccharides (HMOs) present abundantly in breastmilk.^26^ These bacteria have a poor ability to be transmitted horizontally via the environment due to their oxygen sensitivity and lack of spore formation,^27^ and they appear to have evolved to rely mostly on vertical transmission at birth, after which they persist indefinitely.^24^ If such bacteria are lost or fail to colonize at birth, it may take a long time to regain them, making them particularly vulnerable to disturbance.

## Importance of breastmilk

Breastmilk contains > 10 g/l human milk oligosaccharides (HMOs) undigestible by human enzymes. There is a rich diversity of HMOs, with 2ʹfucosyllactose (2ʹFL) and trifucosyllacto-N-hexaose (TF-LNH) being usually the most abundant individual oligosaccharides at 2–3 g/l each in secretor mothers.^28^ The human milk oligosaccharides show great diversity within and between women, the composition varying temporally and according to diet and genotype. As HMOs constitute ca. 20% of all carbohydrates in breastmilk,^28^ and the majority of them are degraded by gut bacteria into short-chain fatty acids,^29^ HMO fermentation represents a significant energy source to the infant. Several species of *Bifidobacterium* and *Bacteroides* have the capacity to ferment human milk oligosaccharides (HMOs),^26^ which very likely explains their success in colonizing the infant gut. While several *Bifidobacterium* species are specialized in oligosaccharide utilization, *Bacteroides* species have the capacity to opportunistically use both diet-derived and host-derived glycans and they employ the same pathways for host mucin and HMO degradation.^26^ On the contrary, HMO-utilizing bifidobacteria do not degrade mucin efficiently and have an advantage in degrading non-mucin-like HMO structures.^26^ In addition to providing substrates for bacterial fermentation, breastmilk is immunologically active, containing immunoglobulins and anti-microbial compounds, which can guide the development of the infant gut microbiota. Consequently, the gut microbiota of exclusively breastfed infants differs from the non-exclusively breastfed in both composition and function.^30^

## Genetic and maternal effects on gut microbiota

Genetic inheritance can essentially be divided into three categories: nuclear genes, which come from both parents, mitochondrial genes, which are inherited from the mother, and microbial genes, which are partly derived from the mother.^20–25^ The father’s role in microbial inheritance is currently not well understood, but a recent report suggests it increases in importance after the first year of life.^24^ Importantly, the inheritance of microbial genes differs from the inheritance of human genes in many ways. Although specific microbes are obtained from the mother during and after birth, other microbes are obtained from the environment, and the composition and abundance of the gut microbiota is not inherited, but affected mainly by environmental factors, such as diet. Nevertheless, the vertical transmission of gut microbes from mother to infant and the nourishing by breastmilk of these maternal microbes is a likely driving force preserving symbiotic relationships between host and microbiota. This would explain some observations on the impact of the host genotype on the microbiota.^31^ As gut microbes are strongly dependent on substrate availability, host genes that affect the glycan composition in the gut, such as those for lactase persistence and the fucosylation of mucin and HMOs, are particularly likely to influence gut microbiota composition.^31–34^ The utilization of HMOs by specific microbes is one example, where the genome of the mother determines the HMO structure of breastmilk and how the infant microbiota respond to breastfeeding.^34^ Another way in which the host genotype may influence the gut microbiota is through the immune system. We found a strong association between host innate immunity genotype, microbiota, and disease severity in pediatric patients with inflammatory bowel disease.^35^

There is relatively little data on the effects of maternal diet and health during pregnancy on infant gut microbiota composition. Maternal stress during pregnancy is associated with reduced abundance of bifidobacteria and lactobacilli and increased abundance of Proteobacteria.^36^ Associations have been found between infant gut microbiota and maternal smoking,^37^ maternal BMI,^38^ maternal asthma,^39^ gestational diabetes,^40^ and maternal diet.^41^ Importantly, the casualties and mechanisms behind these prenatal associations have not been identified, but they suggest that infant physiology, affected by prenatal factors, may partly influence which microbes thrive in the gut. Overall, maternal effects through genetics, prenatal factors, transmission of microbes at birth, and breastfeeding have a strong influence on the early development of the infant gut microbiota.

## C-section and intrapartum antibiotics disrupting microbial colonization

While prenatal factors are associated with specific features of the infant gut microbiota, the most profound effect on the colonization and development of infant gut microbiota is caused by birth mode and perinatal exposure to antibiotics. Several large cohort studies of up to 1000 infants each have found consistent effects of birth mode, especially prominently the low abundance of the normally maternally derived *Bacteroides* and *Bifidobacterium* spp. and increased abundance of pathogens in C-section born infants.^42–46^ This can be explained by the fact that C-section eliminates the contact between maternal fecal microbes and the infant, inhibiting their colonization. The lack of the dominant infant gut microbes, that normally shape the gut environment though their metabolic activity, allows microbes from the hospital environment to colonize the infant.^47^

It is becoming apparent that not all vaginal births result in the same degree of microbial transfer from mother. Little is still known on the effects of different birth interventions, procedures, and practices, with the exception of C-section and intrapartum antibiotic exposure. Maternal exposure to antibiotics during labor impacts the gut microbiota of vaginally born infants with reduced abundance of bifidobacteria^48,49^ and increased abundance of pathogens.^44^ Many of these features are reminiscent of the aberrant microbiota development of C-section born infants.^50^ Importantly, it should be noted, that the main cause of microbiota disruption in C-section born infants is not the antibiotic associated with the surgical procedure but the absence of direct mother to infant contact, since only very few differences have been identified between C-section born infants exposed and not exposed to antibiotic during birth.^51–53^ Postponing the antibiotic administration to after cord clamping is therefore unlikely to help normalize the infant’s gut microbiota.

Antibiotics are usually administered during vaginal birth due to maternal group B streptococcus positivity or suspected infection to prevent transmission of pathogenic microbes to infant. Perhaps not surprisingly, the antibiotic also acts to prevent the transmission of nonpathogenic microbes, resulting in a microbiota composition that resembles that of C-section born infants.^48–50^ Unfortunately, while the beneficial infant-adapted microbes are often highly sensitive to antibiotics and have a poor ability to colonize the infant postnatally, and thus need to be obtained at birth from the mother, many pathogens can be easily acquired from the environment and may be antibiotic resistant. Indeed, the low abundance of bifidobacteria caused by intrapartum antibiotic exposure can lead to increased, rather than decreased, abundance of streptococci in the infant gut.^50^ This is supported by the observation that bifidobacteria can inhibit the growth of streptococci in vitro.^54^

## The case for microbiota restoration

Disrupted gut microbiota development is exceedingly and increasingly common globally. C-sections represent up to 50% of births in certain regions, with additional over 30% of infants exposed to antibiotics during vaginal birth in developed countries.^55,56^ In addition to this, postnatal antibiotic treatments and lack or short duration of breastfeeding are a common and significant disturbance to the infant gut microbiota development.^57,58^ Thus, natural microbiota colonization and development is becoming rare, the consequences of which on public health are not fully understood.

**Figure 1.**
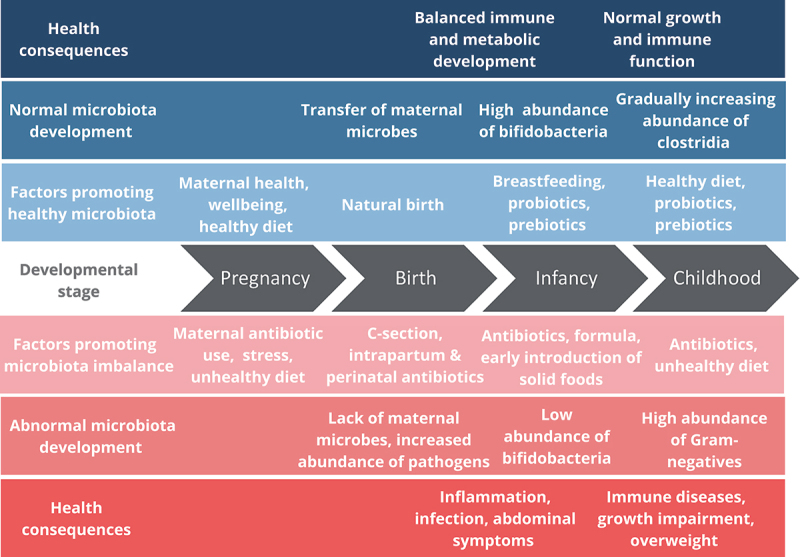
Gut microbiota development and its effects on current and later-life health.

Accumulating evidence indicates that altering the early-life gut microbiota has long-term health consequences (Figure 1). The increased abundance of pathogenic microbes and decreased abundance of beneficial ones may induce inflammation, weaken the gut barrier,^59^ and affect the development of the immune system toward heightened sensitivity. Indeed, signs of pain and distress in infants have been correlated with low abundance of bifidobacteria,^60,61^ symptoms of infant colic can in some cases be alleviated by supplementation with *Lactobacillus reuteri*,^62^ and both C-section born and vaginally born infants that were exposed to cephalosporin antibiotics at birth show increased signs of gastrointestinal discomfort during their first months.^50^

Numerous studies have shown that C-section birth^63–65^ and low abundance of *Bifidobacterium* spp. or high abundance of Proteobacteria^12,66–70^ in early life are associated with the development of chronic immune-related diseases and overweight. The long-term health effects of intrapartum antibiotic exposure have not been thoroughly studied. According to a recent meta-analysis and a large cohort study, intrapartum antibiotics increase the risk of childhood overweight.^71,72^ Prolonged exposure to intrapartum antibiotic has also been found to be associated with increased risk of atopic dermatitis.^73^ Similarly, repeated use of macrolide antibiotics in early life has been linked to increased BMI and risk of asthma in later childhood.^74^ While long duration of breastfeeding is protective against overweight due to the beneficial effects on the gut microbiota, even a single course of antibiotics during the breastfeeding period eliminates the protective effect, strongly indicating a causal role of the gut microbiota in metabolic development.^58^

Gut microbiota is emerging as a potential mediator of the intergenerational transmission of overweight.^75^ Interestingly, the link between maternal BMI and infant gut microbiota appears to be eliminated by C-section birth.^76^ Therefore, one might be tempted to speculate that in some cases eliminating the effect of maternal gut microbiota by either C-section birth or intrapartum antibiotic treatment might be beneficial for the infant. However, C-section birth increases the risk of overweight in the infants of both normal weight and obese mothers, and maternal obesity and C-section birth appear to contribute additively to overweight risk.^75^ Vaginal birth is thus associated with reduced risk of offspring overweight in both normal weight and obese mothers. The overweight risk in infants born by C-section to obese mothers is therefore likely not mediated by transmission of maternal microbiota, but partly by the abnormal microbiota caused by C-section delivery and partly by genetic and environmental and life-style effects.

Preterm infants represent a vulnerable group that are prone to intestinal infections among other complications and are often subject to microbiota-compromising treatments, such as C-section birth, antibiotics, and lack of maternal skin contact and breastfeeding. Their gut microbiota development differs from term-born infants, beginning the normal developmental pattern when they reach the gestational age of term birth,^77^ suggesting that the immature gut may not be able to sustain a normal microbiota prior to that. Because of this, birth mode has a less clear influence on the preterm infant gut microbiota.^77^ Various studies have shown the benefits of supplements of lactic acid bacteria or bifidobacteria in reducing the incidence of necrotizing enterocolitis and mortality in preterm infants, with multispecies products achieving the best outcomes.^78^ In addition, feeding breastmilk has long been known to reduce the incidence of necrotizing enterocolitis.^79^ Clearly, the gut microbiota is extremely important for the health and survival of prematurely born infants. However, restoration efforts should be specifically designed with their unique physiology and vulnerabilities in mind, accepting that term-like gut microbiota may not be biologically realistic or desirable. Because of this, we will focus the following analysis on microbiota restoration in term-born infants.

## Correction of infant gut microbiota after disrupted colonization

A few different microbiota-targeting treatments have been applied with the aim of improving the gut microbiota composition of C-section-born infants: supplementation with specific lactic acid bacteria marketed as probiotics, vaginal seeding, and maternal FMT. In addition to these, exclusive breastfeeding, especially by secretor mothers,^34^ shows significant improvements in microbiota composition compared to formula feeding.^34,80^

Surprisingly few interventions with live bacteria have been reported in term-delivered C-section-born infants. In a large study with over 1000 infants from mothers with a high risk of atopy, a multispecies mixture consisting of *Lactobacillus rhamnosus* GG, *L. rhamnosus* LC705, *Bifidobacterium breve* Bb99, *Cutibacterium freudenreichii* ssp. *shermanii* with additional fructo-oligosaccharides (termed here *Lactobacillus-Bifidobacterium*-FOS supplement), was tested in a randomized placebo-controlled trial.^81^ This intervention was found to reduce the risk of IgE-associated allergy until the age of 5 years in the over 140 C-section delivered infants but not in the around 760 vaginally delivered ones.^81^ This is likely caused by correction of the microbiota, as the gut microbiota of the C-section born infants at 3 months of age was found to be partly normalized by the multispecies treatment.^82^ Among the vaginally born infants, allergy development was likely affected by factors other than the gut microbiota in infancy. These studies demonstrate that infant gut microbiota restoration can have long-term health benefits. In a smaller study with exclusively formula-fed infants, *Lactobacillus reuteri* supplementation modified the gut microbiota of C-section born infants toward the vaginally born composition.^83^ In a recent study, short-term supplementation of C-section born infants with *B. breve* and *L. rhamnosus* for the first two days of life caused a sustained increase in the abundance of lactobacilli, and a non-significant trend of increased bifidobacteria at 1 month.^84^ This suggests that even short-term interventions at the birth hospital may have some benefits.

Lack of exposure to vaginal microbes clearly differentiates C-section born infants from the vaginally born. For this reason, it is easy to speculate that reconstituting the vaginal microbial exposure would restore normal gut microbiota. However, a closer inspection reveals that vaginal microbes are adapted to the vaginal environment, and usually do not find a suitable niche in the infant gut.^25,85^ Three studies have investigated the efficacy of microbiota restoration by inoculating term C-section born infants with maternal vaginal microbes either by swabbing the neonate with vaginal fluids^86,87^ or giving the infant vaginal bacteria orally.^85^ None of the studies have demonstrated a restoration of the normal gut microbiota composition, although one study showed effective seeding of some fecal bacteria.^87^ Interestingly, the study showed evidence indicating long-term effects of vaginal seeding on skin microbiota,^87^ suggesting that vaginal seeding may have benefits outside the gut.

Because the normal inhabitants of the infant gut are fecal microbes obtained from the mother’s gut at birth,^24^ maternal fecal microbiota transfer appears to be the optimal method of neonate gut microbiota restoration. We tested this idea in a pilot study and demonstrated that maternal FMT fully corrected the gut microbiota of C-section – born infants with sustained effects of a single oral administration at birth for at least 3 months.^4^ With carefully screened mothers not carrying known pathogens, the procedure caused no adverse effects on the infants apart from a transient increase in CRP in one infant. Of note, since this study did not alter the mothers’ microbiota or their breastmilk composition, it did not support the hypothesis that the deviating breast milk microbiota in mothers of C-section born infants was involved in the aberrant microbiota development.^88^ In contrast, the study provided further support for the concept that fecal-oral microbiota transfer is the normal way of vertical microbiota transmission.

**Figure 2.**
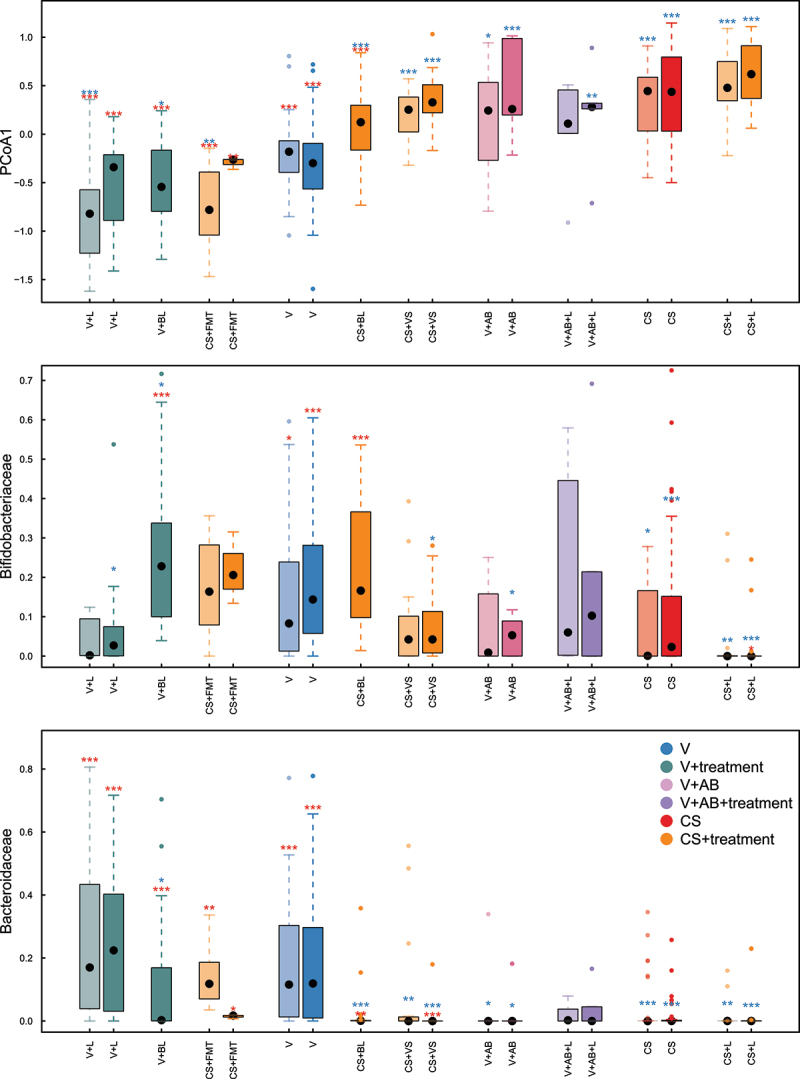
Differences in gut microbiota composition between groups of infants stratified by birth mode and treatment. (a) scores on the first principal component, (b) relative abundance of *bifidobacteriaceae*, (c) relative abundance of *bacteroidaceae*. lighter colors refer to 1-month samples and darker colors to 3-month samples. asterisks indicate the significance of the difference from the vaginally born (“V”) group (blue) and the C-section born (“CS”) group (red), data from refs 4, 50, 82, 85, 86. statistical tests were conducted separately within each age group.

Given the variety of the corrective studies discussed here, we were interested in comparing the efficacy of these interventions in term C-section born infants. We collected data on existing infant gut microbiota restoration studies (mentioned above), including all available data from studies comparing the gut microbiota of untreated term C-section born infants to those subjected to a microbiota-targeting treatment. Unfortunately, data were not available for all of the above-mentioned studies. Hence, we compared the efficacy of maternal FMT,^4^ vaginal seeding from the infant’s mother,^85,86^ and two different types of treatments with live bacteria marketed as probiotics: *Lactobacillus* spp. only^50^ (*Lactobacillus reuteri* or *Lactobacillus rhamnosus* GG given regularly since before week 3) or the *Lactobacillus*–*Bifidobacterium-*FOS supplement that was given daily since birth.^82^ In total, this survey included 132 fecal samples collected at 1 month of age, and 249 fecal samples collected at 3 months (See Supplementary Table S1).Principal Coordinates Analysis of the fecal microbiota (as determined by 16S rRNA amplicon sequencing; see Supplementary Material) of samples taken at 1 month and 3 months revealed that at both time points, birth mode divided the samples along the first principal component, indicating that it was the most significant source of variation in microbiota composition (Figure 2a). Vaginally born and C-section born infants formed distinct microbiota clusters, and vaginally born infants exposed to intrapartum antibiotics had a microbiota composition resembling that of C-section born infants (Figure 2a).

In vaginally born infants not exposed to intrapartum antibiotics, *Lactobacillus* supplementation shifted the microbiota further away from the C-section cluster, but failed to do so in the antibiotic exposed infants (Figure 2a). In C-section born infants, neither vaginal seeding nor *Lactobacillus* supplementation succeeded in shifting the microbiota composition toward the composition of the vaginally born infants (Figure 2a). The *Bifidobacterium-Lactobacillus*-FOS supplement, however, did show a significant restorative effect, although the composition was still different from that of infants born vaginally (Figure 2a). Maternal FMT had the most dramatic effect on microbiota composition, shifting the composition fully to that of the vaginally born infants (Figure 2a).

**Figure 3.**
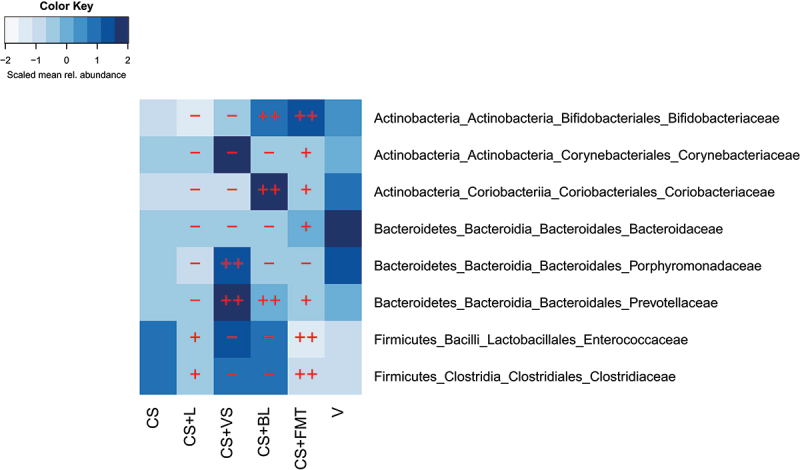
Efficacy of restoration of the microbial families that were significantly different between vaginally born and C-section born untreated infants. – indicates unsuccessful restoration (relative abundance is significantly different from the vaginally born), + indicates moderately successful restoration (no statistically significant difference in relative abundance from the vaginally born group, but difference in mean abundance > 25%), and ++ indicates full restoration (difference in mean relative abundance < 25%, p > .05).

Inspecting the taxonomic differences between the birth groups revealed that the two most abundant bacterial families in vaginally born infants, *Bifidobacteriaceae* and *Bacteroidaceae*, were significantly reduced in C-section born infants (Figure 2b,c). The difference in bifidobacteria was especially clear at 3 months, when bifidobacteria tend to peak in relative abundance^7^ (Figure 2b). Both the bifidobacteria-containing supplement and maternal FMT caused an increase in bifidobacteria, while the lowest relative abundance of *Bifidobacteriaceae* was observed in the *Lactobacillus*-supplemented C-section born infants (Figure 2b). Importantly, the relative abundance of *Bacteroidaceae* was restored only by the maternal FMT treatment (Figure 2c, Figure 3). Of the other families that showed a significant difference between the birth modes, most followed a similar pattern, with the exception of the generally low-abundance *Porphyromonadaceae*, which were restored only by vaginal seeding (Figure 3, Supplementary Figs. 1 and 2). This can be rationalized since this family contains species that are present in the vagina, being implicated in negative pregnancy outcomes, such as preterm birth.^89^ However, it is not clear from the 16S rRNA gene data whether the transferred species are the same as the ones normally present in infant gut. Vaginal seeding also appeared to induce abnormally increased relative abundance of several taxa, such as *Corynebacteriaceae, Staphylococcaceae, Enterococcaceae, Clostridiaceae, Veillonellaceae, Lachnospiraceae*, and *Ruminococcaceae* (Supplementary Fig. 1, 2).

Of all the microbiota-targeting treatments in the CS-born infants, maternal FMT resulted in microbiota composition most closely resembling that of vaginally born infants, while vaginal seeding did not greatly alter the overall composition (Figure 2a). *Lactobacillus*-only supplementation appeared to have minimal benefits in terms of microbiota restoration in CS-born and antibiotic-exposed infants, but the *Bifidobacterium-Lactobacillus-*FOS supplement shifted the microbiota toward the composition as observed in the vaginally born infants (Figure 2). Based on the present analysis, maternal FMT seems the best way to normalize the disturbed microbiota in C-section born infants. Since this experimental treatment was tested in a proof-of-concept trial, there is a need to reproduce this under controlled conditions with a larger sample size. Notably, effects on the immune system development need to be addressed, and a first study in this direction is ongoing (see www.clinicaltrials.gov NCT04173208).

Most of the infants in the analysis were breastfed: 91% at 1 month and 95% at 3 months. Our earlier analysis showed that the beneficial effect of the *Bifidobacterium-Lactobacillus*-FOS treatment in C-section born infants was dependent on breastfeeding.^82^ While breastfeeding alone is insufficient in most cases to restore normal gut microbiota to C-section born infants,^34^ breastfeeding should be considered an essential part of any infant gut microbiota restoration effort. Breastmilk likely plays only a minor role as a source of bacteria to the infant gut, since the composition of breastmilk varies greatly between mothers and often does not include dominant gut microbes.^88^ Through its complex composition, mother’s milk provides both the baby and the existing gut microbiota with the right nutrients to support their growth, development, and symbiosis.

This review focused solely on infant gut microbiota, but the skin and respiratory tract microbiota may play an important role in infant health, as well. Specific gut-targeting treatments are unlikely to be effective in restoring microbial diversity at other body sites. Given that vaginal seeding and maternal FMT will provide the C-section born infant with different types of bacteria, and that naturally infants are exposed to both maternal vaginal and fecal microbes during birth, it will be interesting to see the outcome of an ongoing trial with both vaginal seeding and maternal FMT on infant microbiota and immune health (see NCT03928431).

## Future perspectives

Current results provide promising evidence that gut microbiota restoration of term C-section born infants is feasible and likely has long-term health benefits. However, only a few fairly crude restoration methods have been studied. These should be followed up by studies aimed to identify optimal practices of infant gut microbiota restoration. Generally, to take the field from description to clinically relevant solutions, hence from correlation to causality, hypothesis-driven experimental research in humans is needed. Interventions should be based on clear hypotheses of the biological mechanism that is being tested. Careful research design, longitudinal sample and host data collection, sufficient sample sizes that allow for patient stratification due to individual differences in baseline microbiota, host genetics, or other characteristics, and the utilization of diverse data sources will help to uncover which methods improve the gut microbiota and health in which patient groups. The ecological differences between disrupted microbiota compared to healthy microbiota and the physiological and ecological properties of the key organisms to be restored should inform future restoration studies. For a mechanistic understanding, the gut microbiota should be viewed as an ecosystem consisting of interacting organisms with their unique requirements and characteristics. Importantly, it is not only the relative but also the absolute abundance of microbes that contributes to functional output, and the field would benefit from determining the real amount of gut microbes rather than relying solely on relative abundances.^90,91^ Our recent analysis of early life development based on absolute rather than relative numbers of microbes highlights the need to do so.^50^

## Conclusion

Evidence of the negative health effects of early-life gut microbiota disruption is strong enough to warrant action, especially when weighed against the demonstrated safety of the various microbiota restoration efforts. While the most effective, practical and widely applicable solution for infant microbiota restoration remains to be identified, promising results have been obtained with bacterial products marketed as probiotics and maternal FMT. Considering the scale of the issue – in many regions more than half of infants are exposed at birth to treatments that disrupt natural microbiota colonization – and the potential health consequences, we believe that infant medical care should benefit from addressing the early life gut microbiota and from exploring avenues to preserve and restore the natural biodiversity that is important for a healthy life.

## Supplementary Material

Supplemental Material

## Data Availability

The data that support the findings of this study are available in European Nucleotide Archive (ENA) at https://www.ebi.ac.uk/ena/, reference numbers PRJEB39137, PRJEB48451, PRJEB27325, PRJNA701480, PRJEB10914.
