# Supplementary material for: Infant gut microbiota restoration: state of the art
Source: Gut Microbes. 2022 Sep 10;14(1):2118811. doi: 10.1080/19490976.2022.2118811 (PMC9467569; doi:10.1080/19490976.2022.2118811)
Supplement: Supplemental Material [file KGMI_A_2118811_SM5055.zip › Supplementary information.docx]

Supplementary information

**Infant gut microbiota restoration: state of the art**

Katri Korpela ^1^ & Willem M. de Vos ^1,2^

^1^Human Microbiome Research Program, Faculty of Medicine, University of Helsinki, 00014 Helsinki, Finland

^2^Laboratory of Microbiology, Wageningen University, 6703 WE Wageningen, The Netherlands

**Supplementary methods**

DNA sequences for the 16S rRNA gene were obtained from ENA for the studies that provided the data (4,7,82,86). One study had metagenomic data (85). The 16S sequences were all processed using the same protocol. Forward and reverse reads were merged, and the sequences were quality filtered using Usearch (92). The reads were annotated using Blast (93). The annotated reads were grouped at different taxonomic levels, and the family-level taxonomic tables were used for the analysis. The metagenomic data were processed using MetaPhlan2 (94), which produced relative abundance tables at different taxonomic levels. The family-level data from MetaPhlan were integrated with the 16S-based data manually, as the nomenclature was slightly different between the MetaPhlan taxonomies and Blast taxonomies.

Principal coordinates analysis was conducted in R (95) using the package vegan (96) using Pearson-correlation distances calculated based on log-transformed relative abundance data. Group differences in in relative abundances of bacterial families were analysed using Kruskal-Wallis rank sum test.

**Supplementary tables and figures**

Table S1. Number of samples in different treatment groups by infant age. VD: vaginal delivery; CS: C-section delivery; LAB: *Lactobacillus* spp. supplement, BIF: *Bifidobacterium* spp. supplement; AB: intrapartum antibiotics (standard for CS); M-FMT: maternal faecal microbiota transplantation; M-VS: maternal vaginal seeding.

| Group | 1 month | 3 months | Reference |
| --- | --- | --- | --- |
| Vaginally born (V) | 39 | 65 | (4)(50)(82)(85) (86) |
| Vaginally born with intrapartum antibiotic (V + AB) | 8 | 5 | (50) |
| Vaginally born with intrapartum antibiotic and lactobacillus supplement (V+AB+L) | 11 | 5 | (50) |
| Vaginally born with lactobacillus supplement (V+L) | 11 | 11 | (7) |
| Vaginally born with bifidobacterium-lactobacillus supplement (V+BL) | 0 | 38 | (82) |
| C-section born (CS) | 29 | 64 | All |
| C-section born with lactobacillus supplement (CS+L) | 15 | 15 | (50) |
| C-section born with bifidobacterium-lactobacillus supplement (CS+BL) | 0 | 31 | (82) |
| C-section born with maternal FMT (CS+FMT) | 7 | 3 | (4) |
| C-section born with maternal vaginal seeding (CS+VS) | 18 | 12 | (85) (86) |

Figure S1. Relative abundance of bacterial families at 1 month by group. Asterisks indicate significance of the difference from the vaginally born untreated group (blue) and from the C-section born untreated group (red): * p<0.05, ** p<0.01, *** p<0.001.

Figure S2. Relative abundance of bacterial families at 3 months by group. Asterisks indicate significance of the difference from the vaginally born untreated group (blue) and from the C-section born untreated group (red): * p<0.05, ** p<0.01, *** p<0.001.

**Supplementary references**

92 Edgar RC. Search and clustering orders of magnitude faster than BLAST. Bioinformatics. 2010 Oct 1;26(19):2460-1.

93 Altschul SF, Gish W, Miller W, Myers EW, Lipman DJ. Basic local alignment search tool. Journal of molecular biology. 1990 Oct 5;215(3):403-10.

94 Truong DT, Franzosa EA, Tickle TL, Scholz M, Weingart G, Pasolli E, Tett A, Huttenhower C, Segata N. MetaPhlAn2 for enhanced metagenomic taxonomic profiling. Nature methods. 2015 Oct;12(10):902-3.

95 R Core Team (2019). R: A language and environment for statistical computing. R Foundation for Statistical Computing, Vienna, Austria. URL <https://www.R-project.org/>.

96 Jari Oksanen, F. Guillaume Blanchet, Michael Friendly, Roeland Kindt, Pierre Legendre, Dan McGlinn, Peter R. Minchin, R. B. O'Hara, Gavin L. Simpson, Peter Solymos, M. Henry H. Stevens, Eduard Szoecs and Helene Wagner (2019). vegan: Community Ecology Package. R package version 2.5-4. https://CRAN.R-project.org/package=vegan
