# Supplementary material for: Infant gut microbiota restoration: state of the art
Source: Gut Microbes. 2022 Sep 10;14(1):2118811. doi: 10.1080/19490976.2022.2118811 (PMC9467569; doi:10.1080/19490976.2022.2118811)
Supplement: Supplemental Material [file KGMI_A_2118811_SM5055.zip › supplfig1.pdf]

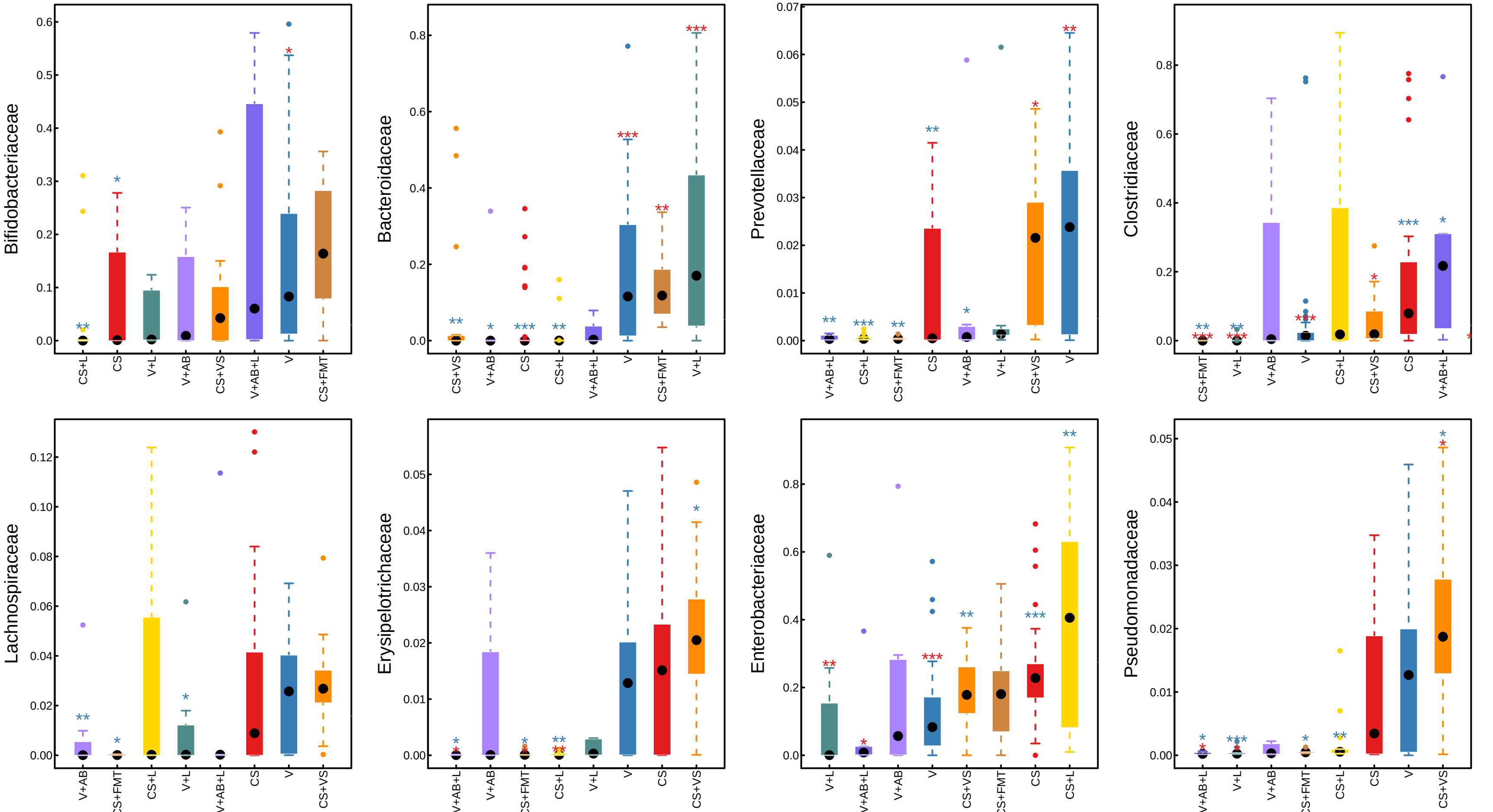

Bifidobacteriaceae
CS+L
CS
V+L
V+AB
CS+VS
V+AB+L
V
CS+FMT
Bacteroidaceae
CS+VS
V+AB
CS
CS+L
V+AB+L
V
CS+FMT
V+L
Prevotellaceae
V+AB+L
CS+L
CS+FMT
CS
V+AB
V+L
CS+VS
V
Clostridiaceae
CS+FMT
V+L
V+AB
V
CS+L
CS+VS
CS
V+AB+L
Lachnospiraceae
V+AB
CS+FMT
CS+L
V+L
V+AB+L
CS
V
CS+VS
Erysipelotrichaceae
V+AB+L
V+AB
CS+FMT
CS+L
V+L
V
CS
CS+VS
Enterobacteriaceae
V+L
V+AB+L
V+AB
V
CS+VS
CS+FMT
CS
CS+L
Pseudomonadaceae
V+AB+L
V+L
V+AB
CS+FMT
CS+L
CS
V
CS+VS
