# Supplementary figures and images for: Infant gut microbiota restoration: state of the art
Source: Gut Microbes. 2022 Sep 10;14(1):2118811. doi: 10.1080/19490976.2022.2118811 (PMC9467569; doi:10.1080/19490976.2022.2118811)

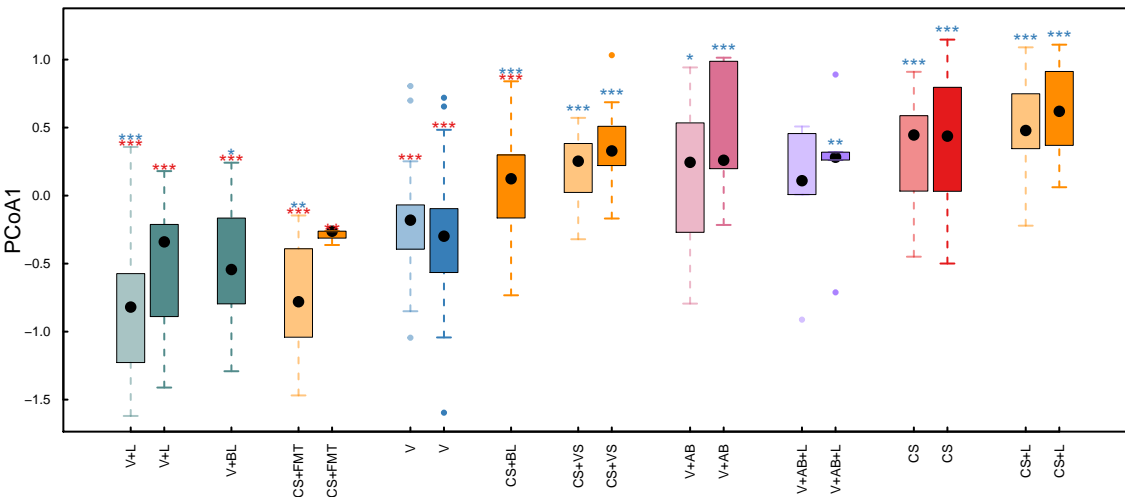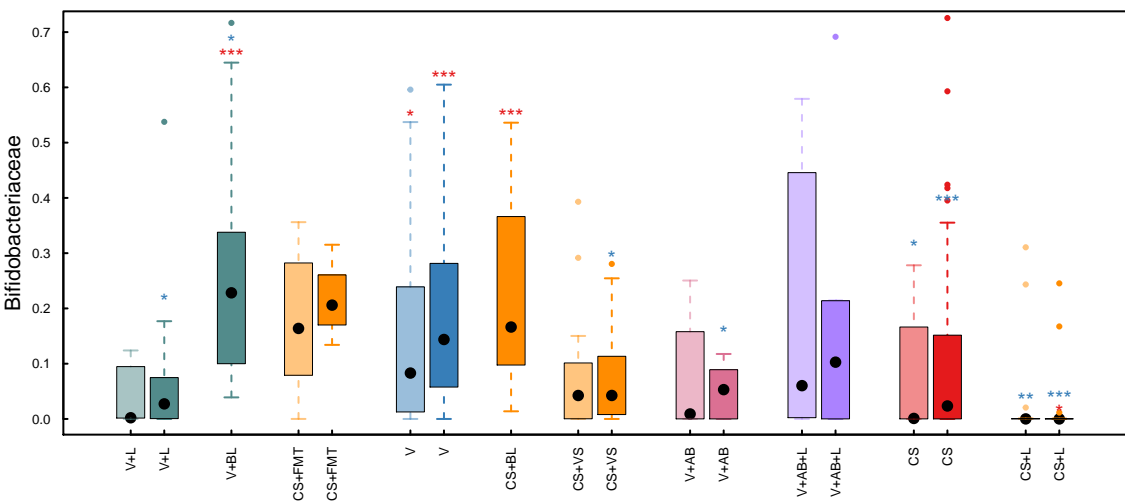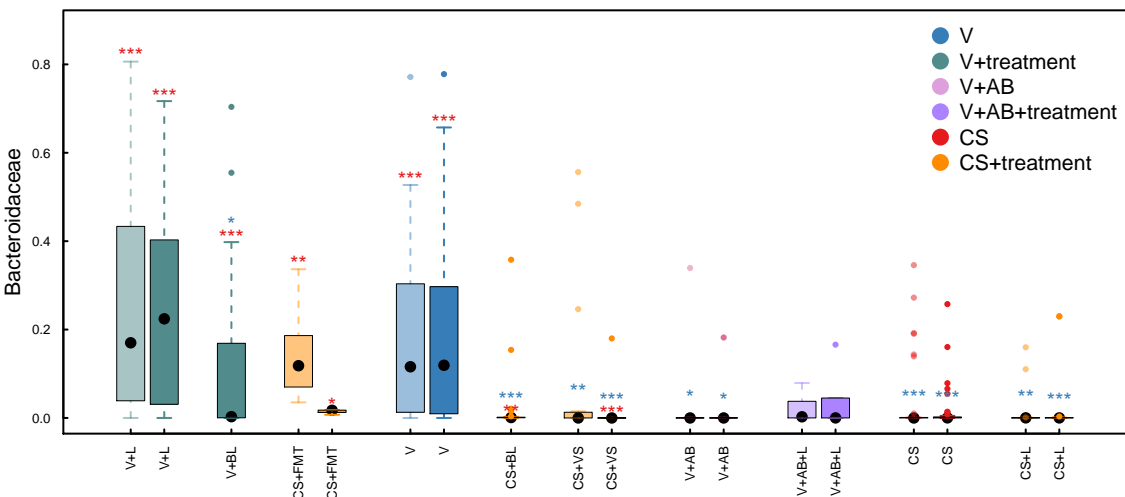

Supplement: Supplemental Material [file KGMI_A_2118811_SM5055.zip › supplfig2.pdf]
